# Asthma: a major pediatric health issue

**DOI:** 10.1186/rr188

**Published:** 2002-06-24

**Authors:** Rosalind L Smyth

**Affiliations:** 1 University of Liverpool, Institute of Child Health, Alder Hey Children's Hospital, Liverpool L12 2AP, UK

**Keywords:** asthma, environmental factors, epidemiology, immunopathogenesis, international data, time trends

## Abstract

The incidence, prevalence, and mortality of asthma have increased in children over the past three to four decades, although there has been some decline in the most recent decade. These trends are particularly marked and of greatest concern in preschool children. Internationally, there are huge variations among countries and continents, as demonstrated by the International Study of Asthma and Allergies in Childhood. In general, asthma rates were highest in English-speaking countries (UK, New Zealand, Australia, and North America) and some Latin American countries (Peru and Costa Rica), and lowest in South Korea, Russia, Uzbekistan, Indonesia, and Albania. There is currently no unifying hypothesis to explain these trends or any associated risk factors. Environmental factors that may lead to asthma include air pollution; genetic factors, the hygiene hypothesis, and lifestyle differences also play potentially causative roles. Asthma may develop as a result of persistent activation of the immune system alone or in combination with physiologic airway remodeling in early childhood. Further studies are needed to confirm this hypothesis.

## Introduction

Increases in both the incidence and prevalence of asthma have been reported in the UK during the past three or four decades [1-3]. People of all ages and backgrounds are affected [3]. The causes of asthma are unknown but may be related to environmental factors, such as indoor and outdoor pollution, lifestyle, diet, and aeroallergens [4,5].

Asthma is an inflammatory reaction in the airways that may result in restricted passage of air into the lungs, which makes normal breathing difficult [3]. The characteristic symptoms of asthma are recurrent wheezing, breathlessness, a feeling of tightness or pain in the chest, and cough. These symptoms affect people in different ways and often are worsened or prolonged by such triggers as house dust mites, dust, pollen, exercise, and smoke.

The present review addresses trends in the incidence, prevalence, and morbidity of asthma in the UK and worldwide, and considers reasons for these trends.

## Incidence

**Figure 1:**
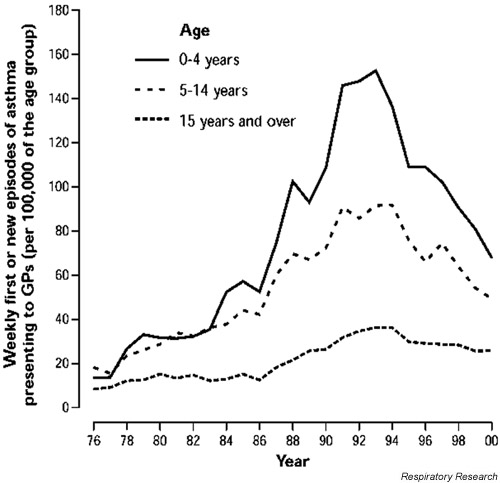
The average weekly incidence of first and new episodes of asthma in patients presenting to general practitioners (GPs) in England and Wales between 1976 and 2000. Reproduced with permission from [3].

Incidence is defined as the number of new cases of asthma that occur in a given period of time [3]. The incidence of asthma attacks or episodes in patients seen by general practitioners in the UK has increased considerably since 1976 (Fig. 1). This increase has occurred in all age groups, with a very large increase in children, particularly preschool children. The incidence in preschool children (0–4 years old) peaked in 1993 at 11 times higher than in 1976. Since 1993, however, the incidence of new episodes of asthma has declined [3,6]. This pattern of an increase followed by a decline was observed in all age groups [6].

Despite the recent decline, the incidence of asthma in the UK currently is considerably higher than it was in 1976, with a weekly incidence of asthma episodes that is substantially higher in adults and several times higher in children [3]. In the year 2000, this accounted for a weekly new case rate (expressed as new cases of asthma per 100,000 of each age group) of approximately 60–70 for preschool children, 40–50 for children aged 5–14 years, and 20–25 for people older than 15 years (Fig. 1).

## Prevalence

Prevalence is defined as the proportion of the population that is affected with asthma at a given time [3]. The prevalence data follow a similar pattern as that observed for incidence. In England, the prevalence of diagnosed asthma for the years 1995–1997 was recorded as being highest (20.7%) in children aged 2–15 years and lowest (10%) in adults older than 45 years. In people aged 16–44 years it was 13.6% [3].

Several investigators have compared the prevalence of asthma and wheeze in particular age groups in the UK over a period of years. For example, Kuehni *et al.*[7] studied the prevalence of asthma and wheezing disorders in young children (aged 1–5 years) between 1990 and 1998. Those investigators reported a significant (*P* < 0.0001) increase in asthma (from 11% to 19%) and current wheeze (from 12% to 26%) in preschool children during this time. In 1978 and 1991, Anderson *et al.*[1] distributed a questionnaire to parents of children aged 7.5–8.5 years. They observed a relative increase in 12-month prevalence of asthma and wheezing. During a similar period, Burr *et al.*[2], who studied children aged 12 years in 1973 and 1988, reported an increase in the prevalence of wheeze in the last 12 months from 9.8% to 15.2%. Lewis *et al.*[8] also reported an increase in prevalence of asthma or wheezy bronchitis in 16-year-old children from 3.8% in 1974 to 6.5% in 1986. Taken together, these data indicate a higher prevalence of asthma and wheezing disorders in children of all ages (1–16 years).

Lewis *et al.*[8] studied possible reasons for this increase in prevalence in these 16-year-old children and found that it was not associated with maternal age, birth order, birth weight, infant breast-feeding, maternal smoking during pregnancy, the child smoking, or father's social class. Those researchers and Kuehni *et al.*[7] suggested that probable causes of asthma were outdoor and indoor pollutants (including allergens and passive exposure to cigarette smoke), diet, obesity, exercise patterns, and a decreased intake of antioxidants such as vitamin C. Alternatively, pulmonary responsiveness to environmental triggers may have changed over the past 25 years [7].

## Ethnic diversity

The rate of asthma in the UK was investigated by Duran-Tauleria *et al*. [9] in different ethnic groups: white children and children of Afro-Caribbean and Indian subcontinent descent. Those investigators reported a high prevalence of asthma in children of Afro-Caribbean descent. This finding is in agreement with that of another report [3], which indicated an approximate 30% prevalence of physician-diagnosed asthma in Afro-Caribbean boys. In comparison, boys of Indian, Pakistani, and Bangladeshi descent had a prevalence rate of less than 20%. Overall, the prevalence of physician-diagnosed asthma in children in the general population of the UK is currently around 20% [3].

## Hospital admissions

The impact of asthma on the health care system in the UK was determined on the basis of hospital admissions (unpublished data). The number of people admitted to hospital for asthma has followed the same trends observed for incidence and prevalence. In 1998 and 1999, the modal duration of stay in hospital was 1 day.

**Figure 2:**
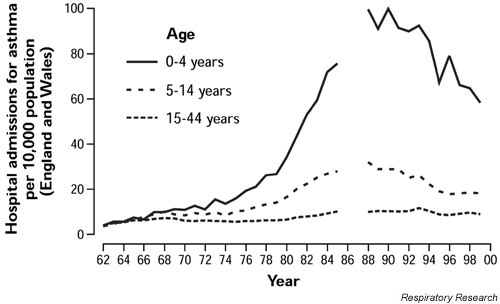
The rate of hospital admissions for asthma by age group from 1962 to 2000. Reproduced with permission from [3].

Between 1962 and 2000, the number of preschool children (newborn to age 4 years) hospitalized for asthma increased considerably (Fig. 2) [3]. This increase began in the late 1970s, peaked between 1988 and 1990, and is currently declining. During this same period children aged 5–14 years underwent a small increase in hospital admissions, whereas people aged 15–44 years maintained the same rate of hospital admissions. In 1989, the asthma hospitalization rate (expressed per 10,000 of the population) was around 90 for preschool children, 30 for older children (aged 5–14 years), and approximately 10 for those aged 15 years or older [3]. Ten years later (1999), hospital admissions for asthma declined, with estimates of 60 per 10,000 for preschool children, 20 for children aged 5–14 years, and 10 for those aged 15 years or older. Nevertheless, the current rate of hospitalization for asthma in preschool children is more than three times higher than that in older children and six times higher than that in adults. These data indicate that preschool children carry the greater burden of asthma, which is causing more medical problems. Some of the increase in rates recorded may be due to improved case ascertainment of asthma in younger children (i.e. children with acute onset of wheeze are more likely to be diagnosed with asthma than in previous decades). However, there is probably also a true rise in the prevalence of asthma in younger children. These data indicate that preschool children are much more likely than older children or adults to develop asthma attacks that are not effectively controlled by reliever medication administered at home.

## Access to health care

Recent changes in primary care in the UK may have been responsible for the improvement in statistics associated with asthma morbidity. These include the introduction of national guidelines for the management of asthma, the introduction of asthma clinics in primary care settings, and improvements in training of staff who care for children with asthma. Asthma clinics in primary care are generally staffed by nurses and primary care physicians who have been trained in asthma management. A number of hospital Accident and Emergency Departments have observation wards attached to them that are staffed by experienced clinicians who closely observe and stabilize the acutely asthmatic child. If there is improvement then the child may be released from the hospital after a relatively short period of observation. Additional strategies to prevent readmission include intensive follow up by nurse practitioners. These changes, in particular the use of clinical nurse specialists or clinical nurse practitioners, have been temporally associated with the decline in hospital admissions [6]. This reduction was particularly evident in young children. It is possible that efforts such as these will continue to decrease hospital admissions in the future.

## Mortality

The number of deaths from asthma has declined in recent years. In the year 2000 asthma accounted for 1500 deaths in the UK, the majority of which were in the elderly [3]. However, in 1999 approximately 25 children and more than 500 adults younger than 65 years died from asthma. These trends provide some reassurance that the increase in hospital admissions in children that has occurred over the past three decades has not been associated with an increase in life-threatening events in this age group.

## International comparisons

The prevalence and severity of asthma in different regions of the world have been studied through the International Study of Allergies and Asthma in Childhood (ISAAC) project [4,5,10]. A simple one-page questionnaire to assess symptoms was given to two groups of children, those aged 13–14 years and those age 6–7 years, in 56 countries with different languages, ethnic composition, and degree of economic development. The older children completed the questionnaire themselves, and parents completed the questionnaire for the younger children. Many study centers used a video questionnaire because it was easier for children to see the symptoms and signs, and thus to determine whether they had experienced them [10].

The sample size appropriate for precise assessment of symptom severity was 3000 per age group per study center [4,10]. For prevalence estimates, a sample size of more than 1000 children per study center was sufficient. Together, data on nearly half a million children in the 13- to 14-year-old age group have been collected.

The data indicate a wide variation in the prevalence of asthma in different parts of the world [4,5]. The self-reported 12-month prevalence of wheezing in children aged 13–14 years ranged from 2.1% in Indonesia to 32.2% in the UK [4]. Similarly, parent-reported 12-month prevalence of wheezing in children aged 6–7 years ranged from 4.1% in Indonesia to 32.1% in Costa Rica. The prevalence was highest (>20%) in English-speaking countries (such as UK, New Zealand, and Australia) and in North America and some Latin American countries, such as Peru and Costa Rica [4]. Countries with the lowest prevalence of asthma (<3%) included South Korea, Russia, Uzbekistan, Indonesia, and Albania [4,10]. Some countries, such as Uzbekistan and South Korea, had a low prevalence of diagnosed asthma but a relatively high prevalence of wheeze [4]. Taken together, the data suggest that there are more cases of asthma in more westernized, affluent countries. In addition, the risk for asthma may be related to environmental factors associated with a modern, Western way of life [2].

In order to investigate further the relationship between asthma and economic deprivation, Stewart *et al*. [11] examined the relationship between the prevalence of wheeze in a 12-month period in 1999 and the 1993 gross national product (GNP) per capita for each country. GNP per capita was used as a measure of the socioeconomic status of a country. Wheeze was the asthma symptom measured. The results indicated a statistically significant (*P* < 0.05) positive association between wheeze and GNP per capita in the 13- to 14-year-old age group, but not in children aged 6–7 years. Those authors concluded that the association between wheezing (asthma) and GNP per capita was moderate to relatively weak. This finding implies that environmental factors other than those related to the wealth of a country may be important in the pathogenesis of asthma in children.

## Environmental factors and asthma

Factors that may lead to asthma include air pollution, genetic influences, and the hygiene hypothesis [4,5]. Air pollutants include indoor agents (i.e. cigarette smoke and house dust mites) and outdoor pollution from motor vehicles [1], as well as aeroallergens [4]. Lifestyle differences, including eating habits, should also be considered [5]. It is possible that asthma is caused by a combination of environmental factors that are influenced by allergens, pathogenic agents, climate, eating habits, lifestyle, and air, water, and/or food pollution [5].

### Air pollution

A link between air pollution, particularly from car exhaust fumes, and asthma was not clearly established in the first phase of the ISAAC study. For example, the prevalence of asthma was low in areas with high levels of air pollution, such as Santiago de Chile, and high in areas with low air pollution, such as New Zealand [5]. The effect of different types of air pollution (indoor and outdoor pollutants) on asthma remains to be determined.

### Genetic factors

The variations in asthma observed in different countries in the ISAAC study cannot be accounted for entirely, or largely, by genetic factors [4,10]. Studies of migrant populations have shown that people who move from one country to another acquire the same asthma prevalence as the host population [5]. For instance, Chinese people living in China have a lower prevalence of asthma than those living in North America. These data indicate that genetic factors are not responsible for differences in asthma between populations, although they may help to explain differences within populations.

### Hygiene hypothesis

The hygiene hypothesis suggests that exposure to infections, particularly in early life, stimulates the immune system to activate T-helper-1 cells, which are associated with antiviral immunity. It proposes that activation of T-helper-1 cells protects against asthma. The information provided by the ISAAC study suggests this may be a local theory, because very high rates of asthma have been observed in developing countries that have low standards of hygiene [5]. For example, in Latin America gastrointestinal parasite infestations and acute viral infections in infancy are common but do not protect against asthma. In fact, some of these countries have as high a prevalence of asthma as in the UK.

## Associations between early wheezing and asthma in later childhood

Several studies were conducted to determine whether wheezing in early childhood predisposed children to asthma later in life [12,13]. Martinez *et al.*[12] followed over 800 infants from birth to ages 3 and 6 years and defined three main patterns of wheezing at these ages. They reported that approximately 20% of these children had wheezing when they were younger than 3 years old but that this had stopped by age 6 years. This pattern was termed 'transient early wheezing'. Approximately 15% of all children did not wheeze until age 6 years (termed 'late-onset wheezing'). The pattern for children (14%) who wheezed at both 3 and 6 years of age was termed 'persistent wheezing'.

Children with transient early wheezing were distinguished from the other groups by lower levels of lung function [12]. This diminished lung function was evident after birth and before the appearance of any lower respiratory tract illness. This factor suggests that congenitally smaller airways may predispose infants to wheezing in early life. At 6 years of age, those children were no longer symptomatic. Transient early wheezing was associated with mothers who smoked, which suggests that maternal smoking may result in smaller airways in their children.

Martinez *et al.*[12] reported that children with persistent wheezing had had normal lung function as infants. Factors associated with persistent wheezing included maternal smoking, maternal asthma, and other atopic diseases. In contrast, late-onset wheezing showed no association with maternal smoking but was associated with maternal asthma, male sex, and rhinitis in the first year of life. As infants, the late-onset wheezing group also had normal lung function. One of the environmental associations that were different in the late-onset wheezing group as compared with the persistent wheezing group was maternal smoking. The investigators concluded that most infants who wheeze are not at increased risk for asthma later in life. In some, however, early wheezing may be related to a predisposition to asthma. Such children may present with activated immune systems that are characterized by elevated serum IgE levels and deficits in lung function.

Children who have respiratory viral infections in infancy may be at risk for respiratory problems later in life. Stein *et al.*[13] studied the association between respiratory syncytial virus (RSV) bronchiolitis in infants and the development of wheezing and asthma in later childhood. Infants were recruited at birth and followed through 6 years of age. At age 6 years, children with previous RSV bronchiolitis had a significantly (*P* < 0.05) increased risk for frequent and infrequent wheeze. From ages 6 to 13 years, the risk for infrequent and frequent wheeze decreased markedly with age, and at 13 years there was no significant difference related to previous RSV bronchiolitis. Those investigators concluded that RSV lower respiratory tract illness was not a risk factor for the development of atopic asthma. However, Martinez [14] suggested that if airway injury, such as may be caused by RSV bronchiolitis, occurs at a time of rapid growth, then this may lead to airway remodeling and the physiologic effect of persistent wheeze.

## Conclusion

There has been a considerable increase in the incidence, prevalence, and mortality of asthma in children in the past three to four decades, with some decline in the most recent decade. These trends are particularly marked and of greatest concern in preschool children. Internationally, there are huge variations from one country and one continent to another. There is currently no unifying hypothesis to explain these trends and associated risk factors. Environmental factors that may lead to asthma include air pollution. Genetic factors, the hygiene hypothesis, and lifestyle differences may also contribute. In addition, these trends and risk factors may differ between populations. It is possible that immunologic events and airway remodeling in early childhood may contribute to the persistence of wheeze and development of asthma. Further studies are needed to confirm this hypothesis.

## Abbreviations

GNP = gross national product; ISAAC = International Study of Asthma and Allergies in Childhood; RSV = respiratory syncytial virus.
